# Ebosin Ameliorates Psoriasis-Like Inflammation of Mice *via* miR-155 Targeting *tnfaip3* on IL-17 Pathway

**DOI:** 10.3389/fimmu.2021.662362

**Published:** 2021-04-26

**Authors:** Weiwei Guo, Fengying Xu, Zhuochen Zhuang, Zhe Liu, Jiming Xie, Liping Bai

**Affiliations:** ^1^ NHC Key Laboratory of Biotechnology of Antibiotics, CAMS Key Laboratory of Synthetic Biology for Drug Innovation, Institute of Medicinal Biotechnology, Chinese Academy of Medical Sciences & Peking Union Medical College, Beijing, China; ^2^ Inner Mongolia Medical University, Inner Mongolia People’s Hospital, Hohhot, China

**Keywords:** psoriasis, ebosin, inflammation, miR-155, *tnfaip3*

## Abstract

Psoriasis is a recurrent autoimmune skin disease with aberrant regulation of keratinocytes and immunocytes. There is no universally accepted single treatment available for psoriasis, and the establishment of a common treatment option to control its signs and symptoms is urgently needed. Here, we found Ebosin, a novel exopolysaccharide isolated from *Streptomyces* sp. 139 by our lab, not only could ameliorate inflammation in LPS-induced keratinocytes through IKK/NF-kapaB pathway, but also attenuate psoriatic skin lesions and reduce inflammatory factors expression in imiquimod (IMQ)-mediated psoriatic mice. Except for inhibiting the expression of epidermal differentiation related proteins, Ebosin significantly increased the percentage of CD4^+^Foxp3^+^CD25^+^ Tregs and decreased CD4^+^IL17A^+^ Th17 cells in psoriatic mice. Furthermore, we demonstrate that Ebosin significantly suppressed the IL-17 signaling pathway *via* A20 (encoded by *tnfaip3*) *in vivo*. As the direct binding of *tnfaip3* to miR-155 has been demonstrated by luciferase reporter assay, and Ebosin has been demonstrated to inhibit miR-155 level *in vitro* and *in vivo*, our study first indicates that Ebosin reduces inflammation through the miR-155-*tnfaip3*-IL-17 axis and T cell differentiation in a psoriasis-like model. Thus, we conclude that Ebosin can act as a promising therapeutic candidate for the treatment of psoriasis.

## Introduction

Psoriasis is a chronic autoimmune disease characterized by epidermal hyperproliferation, inflammatory cell infiltration and vascular proliferation in dermis (1), which affects 2% to 3% of the world population (2). A portion of psoriatic patients could develop into psoriatic arthritis, as well as various complications with an increasing risk, such as hyperlipidemia, cardiovascular disease, diabetes, rheumatoid arthritis, and cancer (3–6). Psoriatic patients are generally treated with glucocorticosteroids and/or conventional immunosuppressants, which could temporarily alleviate symptoms, whereas result in serious side effects (7, 8). In recent years, drugs for psoriasis have been screened as target suppression of IL-17A, IL-23, and Janus kinase (JAK), such as ustekinumab, ixekizumab, and tofacitinib, which are well effective but expensive (9–13).

The occurrence of psoriasis is related to various factors, such as genetic and environmental factors, metabolic disorders, and immunity. So far, its exact pathogenesis remains unclear, but it is generally believed that T cell-mediated immunity plays an important role in its development. Meanwhile, the imbalance between the pro-inflammatory Th17 cells and the anti-inflammatory Treg cells has caused a variety of autoimmune diseases, including psoriasis (14). IL-17 produced by Th17 cells has been shown to play a central role in psoriasis, which induces the production of proinflammatory chemokines, cytokines, and antimicrobial peptides in keratinocytes (15). Tumor necrosis factor alpha induced protein 3 (TNFAIP3/A20) and TNFAIP3 interacting protein 1 (TNIP1) have been accepted as the related candidate genes for psoriasis (16–18). Act1 is an essential adaptor molecule in IL-17–mediated signaling which could mediate the lysine-63–linked ubiquitination of tumor necrosis factor receptor-associated factor 6 (TRAF6) as a U-box E3 ubiquitin ligase (19). That is, IL-17 acts through Act1 and TRAF6 to mediate the downstream NF-κB signaling pathway (20).

MicroRNA (miRNA) is a small non-coding RNA molecule in plants, animals, and some viruses containing about 22 nucleotides. It plays a key role in regulating RNA silencing and posttranscriptional gene expression (21). miRNA participates in a variety of biological procedures, and its expression disorders could result in human diseases, such as cancer, inflammations, and immune abnormalities (22, 23). As with psoriasis, miR-155 has been found to be highly expressed in psoriatic and various autoimmune diseases patients. The level of miR-155 in skin has been demonstrated to have a significantly positive correlation with PASI score and IL-17A content (24). Although several miRNAs have been identified as positive or negative regulators in the regulation of psoriasis, the detailed mechanism needs to be further illustrated.

Ebosin, a novel exopolysaccharide composed of rhamnose, fucose, arabinose, mannose, xylose, glucose, galactose, and galacturonic acid, was isolated from the supernatant of the fermentation culture of *Streptomyces* sp. 139 (25). Previous studies have shown that Ebosin significantly suppresses the development of rat collagen-induced arthritis *in vivo* (26), and inhibits IL-1β–induced MAPK and NF-κB signaling pathway in rat fibroblast-like synoviocytes (FLSs) *in vitro* (25). Interestingly, we found that Ebosin could reduce IFNγ-induced CXCL-9 level in HaCaT cells, which is a key evaluated indicator for psoriasis.

In this study, we found that Ebosin could ameliorate psoriatic skin lesions, improve skin immunopathology, and reduce inflammatory factors expression in imiquimod (IMQ)-mediated psoriasis in a mouse model. Combined with our RNA-seq results on LPS-induced inflammatory HaCaT cell models, we concluded that Ebosin is effective on psoriasis through IL-17 signaling pathway and T cells regulation targeted by miR-155.

## Materials and Methods

### Isolation of Ebosin

*Streptomyces* sp. 139 producing Ebosin was isolated from a soil sample in China and kept in the China General Microbiology Culture Collection Center (No. 0405). The strain was cultured at 28°C for 96 h in TSB medium supplemented with 5 mM MgCl_2_ and 0.5% glycine or fermentation medium. Ebosin was isolated from the supernatant of *Streptomyces* sp. 139 fermentation culture using the previously described protocol (27).

### Cell Lines and Culture

The HaCaT and CCC-ESF-1 cell lines were purchased from The National Experimental Cell Resource Sharing Platform (Beijing, China). The cells were cultivated in Minimum Essential Medium with Eagle’s Balanced Salts (MEM-EBSS; Hyclone, Solarbio, Beijing, China) containing 10% fetal bovine serum (FBS; Gibco Life Technologies, Carlsbad, CA, USA), and were incubated at 37°C with 5% CO_2_. Cells were treated with 10 μg/ml lipopolysaccharide (LPS; Beyotime, Shanghai, China) and series concentration of Ebosin for 24 h. Ebosin was prepared as previously described (25) and was stored at −20°C.

### Cell Viability

HaCaT and CCC-ESF-1 cells were seeded in a 96-well plate at a density of 3×10^4^ cells/ml, and were incubated with Ebosin at concentrations ranging from 0 to 100 μg/ml within 36 h. At the end of exposure, 10 μl MTT (5 mg/ml) was added to each well and cells were incubated at 37°C for 4 h. The media was removed, and 100 μl DMSO was added into each well to dissolve the formazan crystals. Cell viability was detected at 490 nm by Victor X5 multi-label microplate detector (PerkinElmer, MA, USA) and was calculated with a negative control as 100%.

### Experimental Animals

Imiquimod cream (IMQ, 5%) was obtained from Sichuan Mingxin Pharmaceutical Co., Ltd. (Sichuan, China). Methotrexate (MTX) was obtained from SPH Sine Pharmaceutical Laboratories Co., Ltd. (Shanghai, China). Male BALB/c mice of bodyweight 18 to 22 g were purchased from Beijing HFK Bioscience Co., Ltd, allowed to acclimate to a new SPF surrounding (temperature: 22 ± 2°C, humidity: 40−60%, light/dark cycle: 12 h) for 1 week, with food and water supplied *ad libitum*. Animal experimental protocols were performed under NO. IMB-20191025(D3) according to the Chinese National Guidelines for the Care and Use of Laboratory Animals and approved by the Animal Experimental Ethics Committee of Institute of Medicinal Biotechnology, Chinese Academy of Medical Sciences & Peking Union Medical College.

### IMQ-Induced Psoriasis-Like Mouse Model and Animal Treatment

BALB/c mice were randomly divided into six groups, including control group, vehicle group, MTX group (1 mg/kg), Ebosin low-dose group (Ebosin-L, 200 mg/kg), Ebosin medium-dose group (Ebosin-M, 400 mg/kg) and Ebosin high-dose group (Ebosin-H, 800 mg/kg). The back hair of all mice was shaved in an area of 2 cm × 3 cm. All groups except the control group were topically administered with a dose of 62.5 mg IMQ scribbled on the back skin of mice for seven consecutive days. MTX and Ebosin groups were orally administered at a dosage of 1 and 200/400/800 mg/kg/d 7days in advance, and for 14 consecutive days respectively. Mice were sacrificed on the seventh day of IMQ-administration, and the skin, spleens were collected for further analyses.

### Evaluation of the Severity of Psoriatic Skin Lesion

The thickness of skin was measured by callipers. The severity of skin lesion was evaluated by Psoriasis Area and Severity Index (PASI) scored ranging from 0 to 4, which comprises the parameters of skin erythema, scaling, bulge, and thickness. The value of 0, 1, 2, 3, and 4 represents the severity of none, slight, moderate, marked, and severe, respectively. The average score of each group was calculated to evaluate the severity of psoriatic skin lesion.

### Histological Analysis and Immunohistochemistry

Back skin samples of the mice were fixed in 4% neutral paraformaldehyde for 24 h and were then embedded in paraffin. The paraffin block was cut into 3 μm slices. For H&E staining, the slices were stained with 3′-diaminobenzidene (DAB, Sigma-Aldrich) and were counterstained by hematoxylin. For immunohistochemistry staining, slices were incubated with primary monoclonal anti-CD3 (Abcam, ab5690), anti-CD8 (Abcam, ab237723) or anti-Ki67 (Abcam, ab15580) antibody at a dilution of 1:200 at 4°C overnight, then with secondary antibody HPR-anti-Rabbit IgG (CST). The specificity of the primary antibodies was tested by substituting isotype-matched antibodies (Abcam, ab171870 for CD3 and Ki67; Abcam, ab172730 for CD8). Slices were imaged at a magnification of 100×, and the integrated optical density (IOD) of CD3, CD8, and Ki67 was measured using LAS X software (Leica).

### Flow Cytometric Analysis

Spleen cells were prepared and stained for surface markers with anti-CD4-FITC (1:100, BD Pharmingen, 557397) and anti-CD25-PerCP-Cy5.5 (1:250, BD Pharmingen, 551071) separately. To determine intracellular Foxp3 and IL-17A expression, cells were fixed and permeated by Transcription Factor Buffer Set (BD Pharmingen, 562574), and were then stained with anti-Foxp3-PE (1:250, BD Pharmingen, 563101) or anti–IL-17A-Alex647 (1:250, BD Pharmingen, 560184). The frequency of CD4^+^CD25^+^Foxp3^+^ Tregs and CD4^+^IL17A^+^Th17 cells were finally analyzed through FACSCalibur (BD Biosciences).

### Quantitative Real-Time Reverse Transcription PCR (RT-PCR)

Total mRNA was extracted from cells and skin tissues with TRIzol Reagents (Trans, Beijing, China), and was transcribed into cDNA with TransScript One-Step gDNA Removal and cDNA Synthesis SuperMix (Trans, Beijing, China) according to the manufacturer’s introduction. The amplification protocol consisted of an initial step of 30 s at 94°C followed by 40 cycles of 5 s at 94°C and 30 s at 60°C on a BioRad CFX96 (BioRad, USA) using PerfectStart Green qPCR SuperMix (Trans, Beijing, China). The relative expression levels were normalized to *gapdh* level using the 2^−ΔΔCT^ method. The primer sequences are listed in **Table S1**.

### miRNA Determination

For quantification of various miRNA levels, miRNA was extracted with EasyPure miRNA Kit (Trans, Beijing, China) and was transcribed into cDNA with TransScript miRNA First-Strand cDNA Synthesis SuperMix (Trans, Beijing, China). The amplification of miRNA was performed with the specific primers shown in **Table S2** on a BioRad CFX96 (BioRad, USA) under the standard conditions. The relative miRNA levels were determined by normalizing to *U6* using the 2^−ΔΔCT^ method.

### Western Blot

Total protein samples in cells and skin tissues were prepared by RIPA buffer on ice-bath for 30 min. The supernatant was collected after centrifugation at 12,000 rpm for 10 min at 4°C, and the protein concentration was measured by BCA protein assay kit (Applygen, Beijing, China). 30 μg protein of each sample was loaded to an 8% SDS-PAGE gel and was transferred to a PVDF membrane. The PVDF membranes were blocked with 5% (w/v) skimmed milk powder in TBST for 2 h, and were subsequently incubated with primary anti-phospho-IKKα/β (#2697, Cell Signaling Technology, Boston, USA), anti-IKKβ (#8943, Cell Signaling Technology, Boston, USA), anti–phospho-IκBα (#2859, Cell Signaling Technology, Boston, USA), anti-IκBα (#4812, Cell Signaling Technology, Boston, USA), anti-phospho-NF-κB p65 (#3033, Cell Signaling Technology, Boston, USA), anti-NF-κB p65 (#8242, Cell Signaling Technology, Boston, USA), anti-A20 antibodies (#5630, Cell Signaling Technology, Boston, USA) (1:1000 for CST antibodies) and anti-Act1 (sc-398161, Santa Cruz Biotechnology, CA, USA), anti-TRAF6 antibodies (sc-8409, Santa Cruz Biotechnology, CA, USA) (1:200 for SC antibodies) at 4°C overnight. Then the membranes were washed using TBST and incubated with secondary HPT-conjugated goat anti-rabbit or anti-mouse IgG antibody (1:10000) for 1 h at room temperature. The blots were detected by an enhanced chemiluminescence method on a Bio-Rad Gel imaging system and were analyzed by Image J software.

### Cell Transfection

To detect the effect of miR-155 on HaCaT cells, we synthesized miR-155 mimic and inhibitor through Ribobio Co., Ltd. (Guangzhou, China). HaCaT cells transfection was conducted by Lipofectamine 3000 Reagent (Invitrogen, NY, USA) following the manufacturer’s instruction. The miRNA sequences (5′-3′) used in this procedure were listed as follows. miR-155-3p mimic: CUCCUACAUAUUAGCAUUAACA. miR-155-3p inhibitor: UGUUAAUGCUAAUAUGUAGGAG. After being transfected with miR-155 mimic/inhibitor or a negative control (NC) (50 nmol/L), the cells were incubated at a 37°C and 5% CO_2_ incubator for 6 h, and were then incubated for 48 h in substituted fresh MEM containing 10% FBS.

### Luciferase Reporter *tnfaip3* Expression Assay

To measure luciferase activity of *tnfaip3*, the wild type and mutant 3′ UTR sequence of *tnfaip3* were linked in the psi-CHECK2 plasmid (Promega). The HaCaT cells were co-transfected with miR-155-3p mimic or NC and *tnfaip3*-3′ UTR WT or MUT luciferase plasmids by the Lipofectamine 3000 transfection agent in a 96-well plate. Fresh media with 10% FBS was replaced after 6 h transfection. Luciferin intensity was measured using Dual-Luciferase Reporter Assay System (Promega, WI, USA) after 48 h-incubation by Victor X5 multi-label microplate detector (PerkinElmer, MA, USA).

### Statistical Analyses

Data was presented as the mean ± SD of at least three replicants and was analyzed by GraphPad Prism 7 software. One-way ANOVA was applied to statistical comparisons between groups, and *p* < 0.05 was considered to be with significance.

## Results

### Ebosin Ameliorated LPS-Induced Cell Injury

**Figure 1:**
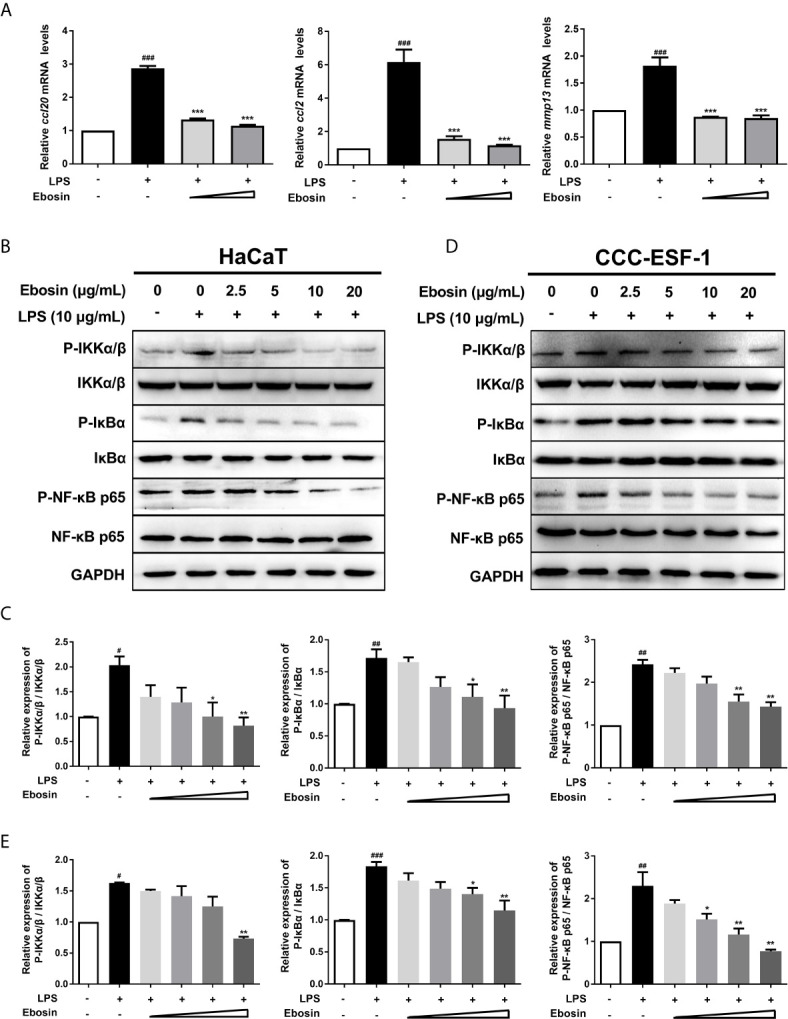
Effects of Ebosin on LPS-induced HaCaT and CCC-ESF-1 cells. **(A)** RT-PCR analyses of genes in HaCaT cells, including *ccl-20*, *ccl-2*, and *mmp13* (mean ± SD, n = 3). **(B, C)** Western blotting analyses for proteins on IKK/NF-κB signaling pathway in HaCaT cells. **(D, E)** Western blotting analyses for proteins on IKK/NF-κB signaling pathway in CCC-ESF-1 cells. ^#^ *P* < 0.05, ^##^ *P* < 0.01, ^###^ *P* < 0.001 vs. control; *P < 0.05, **P < 0.01, ***P < 0.001 vs. LPS-induced group.

Ebosin was detected with no cytotoxicity on HaCaT cells ranging from 0 to 100 μg/ml within 36 h (**Supplementary Figures S1A, B**). qRT-PCR was performed to measure the expression of inflammatory cytokines in HaCaT cells (**Figure 1**). The mRNA levels of *ccl2*, *ccl20*, and *mmp13* induced by LPS-stimulation were significantly decreased upon treatment with 10 μg/ml Ebosin. The key phosphorylated proteins on IKK/NF-κB signaling pathway, including phospho IKKα/β, IκBα, and P65, were reduced by Ebosin in HaCaT (**Figures 1B, C**) and CCC-ESF-1 (**Figures 1D, E**).

RNA-Seq of HaCaT cells with treatment of LPS with or without Ebosin was carried out. Different expressed genes were exhibited in **Table S3**, and functions of different expressed genes were analyzed by KEGG (**Supplementary Figures S2A, B**) and GO (**Supplementary Figures S2C, D**) enrichment. Considering enrichment score, gene numbers, and p-value, the encoding genes of A20, CC- and CXC-chemokines and various cytokines related to IL-17 signaling pathway were considered to be differently expressed (fold change>2 or fold change<0.5, p-value<0.05).

### Ebosin Ameliorates Skin Lesion in IMQ-Induced Psoriatic Mice

**Figure 2:**
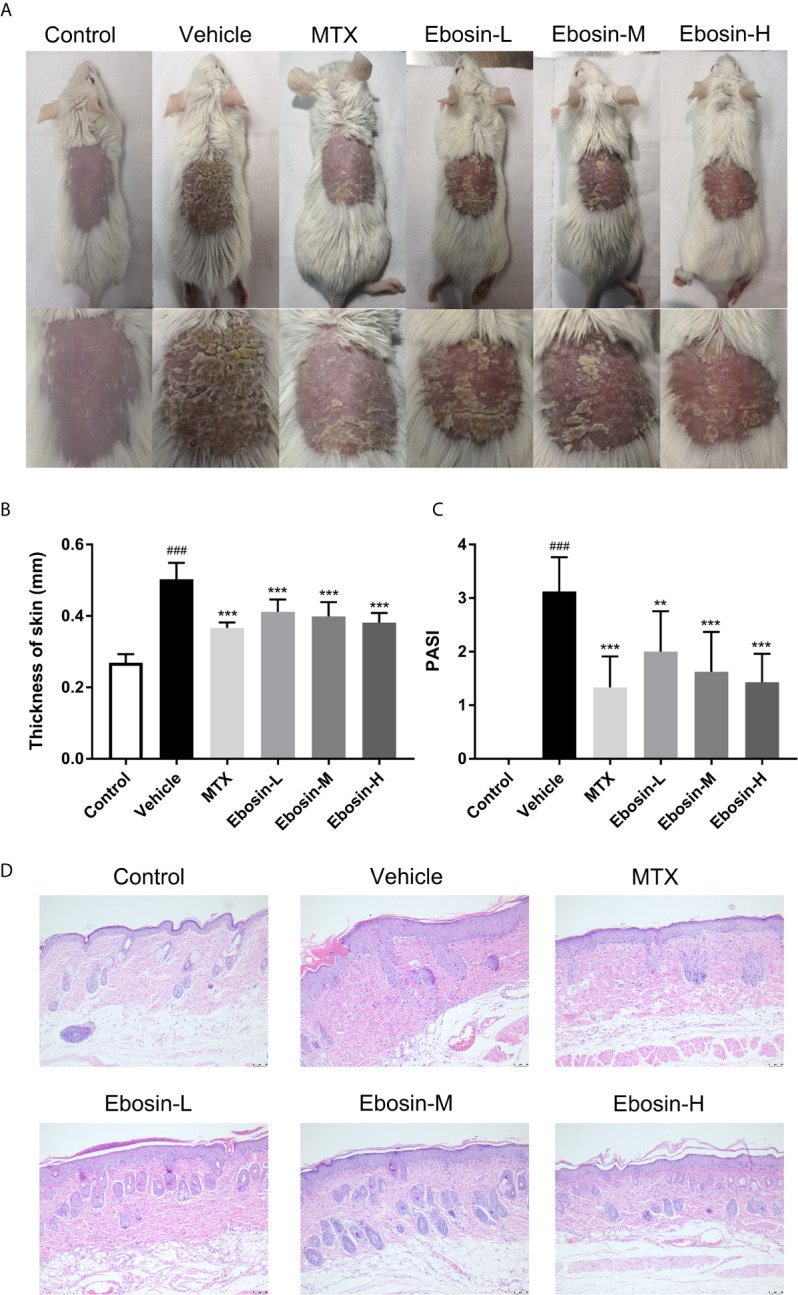
Ebosin ameliorates skin lesion in IMQ-induced psoriatic mice. **(A)** The representatives of photos of dorsal skin in IMQ-induced psoriasis-like mice 7 days after IMQ-treatment without or with Ebosin. **(B)** The thickness of dorsal skin (mean ± SD, n = 6). **(C)** The PASI scores of the skin lesion in IMQ-induced psoriasis-like mice (mean ± SD, n = 6). **(D)** H&E staining of the skin of control or IMQ-induced psoriasis-like mice after treatment without or with Ebosin (scale bar is 50 μm). ^###^ *P* < 0.001 *vs.* control; ^**^ *P* < 0.01, ^***^P < 0.001 *vs.* IMQ-induced vehicle group.

The model of IMQ-induced psoriasis-like mice was used to evaluate the effect of Ebosin on psoriasis. Compared with no skin lesion and sign of inflammation within the control group, the symptoms of psoriasis-like lesions, like scaling and thickening, were deteriorated after 7-day IMQ treatment in vehicle. As expected, treatment with MTX recovered the overall skin lesion. Excitingly, treatment with Ebosin reduced scaling, thickening, and erythema in a dose-dependent manner (**Figure 2A**). The dosage of Ebosin did not cause any change on weight, while the skin thickness and PASI were reduced by Ebosin treatment (**Figures 2B, C**). There were significant psoriatic lesions including epidermal hyperplasia, hyperkeratosis, and acanthosis after IMQ treatment viewed from H&E staining. While the epidermal hyperplasia was significantly alleviated after treatment with Ebosin, high dose of Ebosin exhibited slight advantages with MTX (**Figure 2D**).

### Ebosin Inhibits CD3^+^ and CD8^+^ T Cell Infiltration and Epidermal Hyperplasia in Skin of Psoriatic Mice

**Figure 3:**
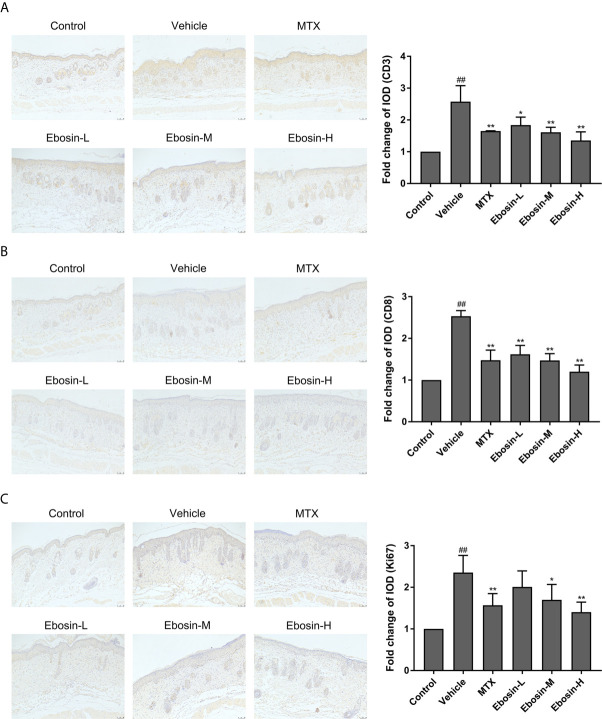
Ebosin inhibits CD3^+^ and CD8^+^ T cell infiltration and epidermal hyperplasia in skin of psoriatic mice (scale bar is 50 μm). **(A)** Immunohistochemical images of CD3 staining (magnification: 100×) of dorsal skin and the relative IOD of control (mean ± SD, n = 3). **(B)** Immunohistochemical images of CD8 staining (magnification: 100×) of dorsal skin and the relative IOD of control (mean ± SD, n = 3). **(C)** Immunohistochemical images of Ki67 staining (magnification: 100×) of dorsal skin and the relative IOD of control (mean ± SD, n = 3). ^##^ *P* < 0.01 *vs.* control; ^*^ *P* < 0.05, ^**^P < 0.01 *vs.* IMQ-induced vehicle group.

Psoriasis is an autoimmune disease mainly mediated by CD3^+^ T cells, and CD8^+^ T cells are known to accumulate in the epidermis of psoriatic skin (28). Psoriasis-like skin inflammation and epidermal infiltration by CD8+ T cells are some of the earliest and most severe features (29). Here CD3 and CD8 expression in skin samples were detected by immunohistochemical assay (Isotype Control was shown in **Supplementary Figure S3**). CD3 and CD8 expression were significantly increased after IMQ treatment. In addition, significant decreases of CD3 and CD8 expression were observed in mice treated with MTX, media-dose of Ebosin (Ebosin-M) and high-dose Ebosin (Ebosin-H) compared to the mice of vehicle group (*p* < 0.01, **Figures 3A, B**), indicating that Ebosin alleviated CD3^+^ and CD8^+^ T cell infiltration in the psoriatic skin.

Ki67, as an indication of cell proliferation, was demonstrated to increase in IMQ-treated mice, and significantly decrease in MTX and Ebosin-M, Ebosin-H groups by immunohistochemical staining (*p* < 0.01 vs vehicle group, **Figure 3C**).

### Ebosin Modulates CD4^+^CD25^+^Foxp3^+^ Tregs and CD4^+^IL17A^+^ Th17 Cells in Psoriatic Mice

**Figure 4:**
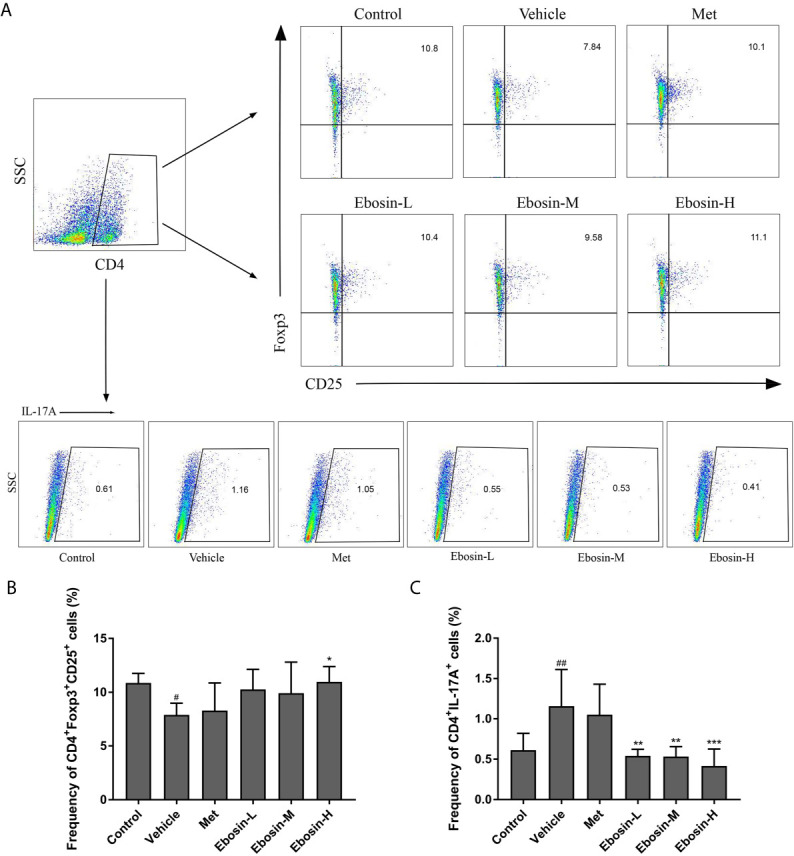
Ebosin modulates CD4^+^CD25^+^Foxp3^+^ Tregs and CD4^+^IL17A^+^ Th17 cells in psoriatic mice. **(A)** Flow cytometric analysis of Treg and Th17 cells in spleen of each group. **(B)** The percentage of CD4^+^CD25^+^Foxp3^+^ Tregs in spleen. **(C)** The percentage of CD4^+^IL17A^+^ Th17 cells in spleen. All data were presented as mean ± SD; n = 6. ^#^ *P* < 0.05, ^##^ *P* < 0.01 *vs.* control; ^*^ *P* < 0.05, ^**^P < 0.01, ^***^P < 0.001 *vs.* IMQ-induced vehicle group.

To evaluate whether Ebosin alleviate psoriasis by inducing regulatory T cells (Treg), spleen cells were isolated and analyzed using a flow cytometer. As shown in **Figure 4**, IMQ-treatment significantly decreased the percentage of CD4^+^Foxp3^+^CD25^+^ regulatory cells compared with control group, and high-dose of Ebosin increased the percentage of Tregs (*p* < 0.05). In addition, CD4^+^IL17A^+^ T cells (Th17) was significantly increased after IMQ treatment and decreased by serial doses of Ebosin (all *p* < 0.01), suggesting that Ebosin hindered the development of Th17 in psoriatic mice. However, there was no significant difference of Tregs and Th17 cells between MTX and vehicle group.

### Ebosin Inhibited the Inflammatory Response and Related Signaling

**Figure 5:**
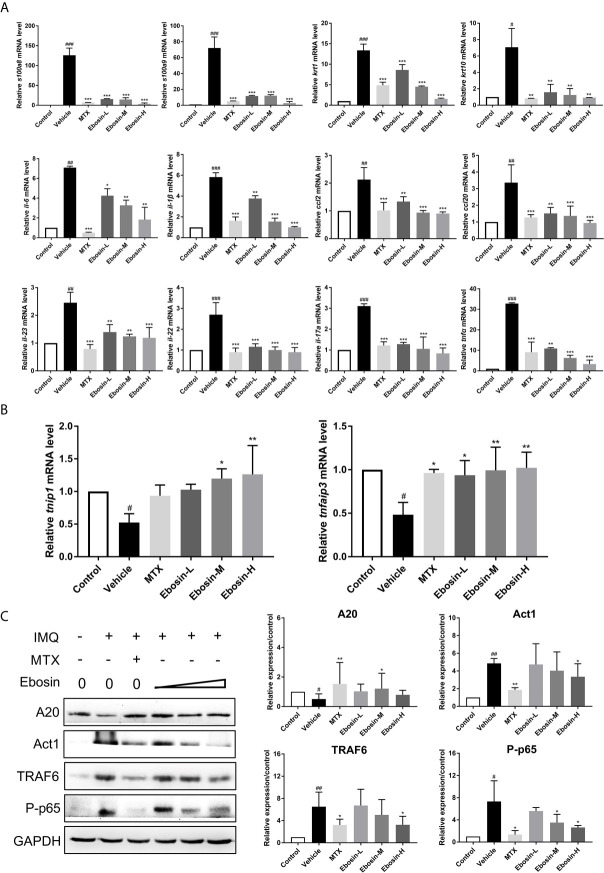
Ebosin inhibited the inflammatory response and IL-17 signaling pathway. **(A)** RT-PCR analyses of cytokines in psoriasis-like mice without or with Methotrexate (MTX) and Ebosin. **(B)** RT-PCR analyses of *tnfaip3* and *tnip1* in psoriasis-like mice without or with MTX and Ebosin. **(C)** Western blotting analyses for proteins on IL-17 signaling pathway in psoriasis-like mice without or with MTX and Ebosin, including A20, Act1, TRAF6, and phosphor-p65. All data were presented as mean ± SD; n = 3. ^#^ *P* < 0.05, ^##^ *P* < 0.01, ^###^ *P* < 0.001 *vs.* control; ^*^ *P* < 0.05, ^**^P < 0.01, ^***^P < 0.001 *vs.* IMQ-induced vehicle group.

The effect of Ebosin on the expression of encoding genes of epidermal differentiation related proteins, cytokines, and chemokines in the skin samples were detected by qRT-PCR. S100A8 and S100A9 are two proteins necessary for normal epidermal differentiation and activation of macrophages (30). Keratin 1 (KRT1) and keratin 10 (KRT10) are key proteins associated with terminal differentiation of keratinocytes. The levels of *s100a8, s100a9*, and *krt1, krt10* were enhanced in vehicle group, and were reduced after administration of MTX and Ebosin (**Figure 5A**, *p* < 0.01). The levels of *il-23, il-22, il-17a, il-6, il-1β, tnfα*, and *ccl2, ccl20* were significantly increased following IMQ treatment (*p* < 0.01, **Figure 5A**). Administration of MTX and Ebosin, especially at high dose, significantly reduced the mRNA levels of encoding genes of cytokines and chemokines mentioned above (*p* < 0.05). More importantly, *tnfaip3* and *tnip1*, two key genes regulating NF-κB signaling in psoriasis, were significantly inhibited by IMQ and were recovered by media & high dose Ebosin (**Figure 5B**, *p* < 0.05).

Ebosin targeting A20 (coded protein of *tnfaip3*)/NF-κB in IMQ-induced psoriatic mice were further investigated by Western blot analysis of skin samples. As shown in **Figure 5C**, A20 protein was significantly diminished after being induced by IMQ, and evoked following Ebosin administration. As expected, TRAF6, Act1, phospho p65 expressions were notably increased by IMQ and attenuated by Ebosin in a dose dependent manner, indicating that Ebosin exerts antipsoriatic effects through regulating A20/NF-κB signaling pathway. However, it is still unclear how Ebosin acts with *tnfaip3* (A20). Our next focus is on miRNAs.

### Effect of Ebosin on miRNA-155 Targeting *tnfaip3*

**Figure 6:**
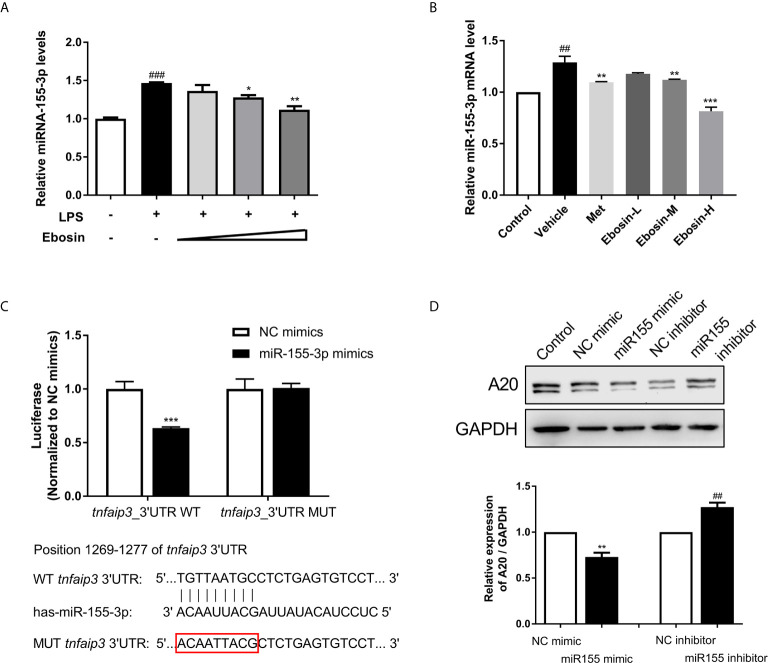
Effect of Ebosin on miRNA-155 targeting *tnfaip3*. **(A)** RT-PCR analyses of miR-155 in LPS-induced HaCaT cells without or with Ebosin. **(B)** RT-PCR analyses of miR-155 in skin of IMQ-induced psoriasis-like mice without or with MTX and Ebosin. **(C)** Schematic view of miR-155 putative targeting site in the wild type (WT) and mutant (Mut) 3′-UTR of *tnfaip3*, and luciferase reporter analysis to confirm the binding between miR-155 and *tnfaip3*. **(D)** The protein expression level of A20 in HaCaT cells introducing miR-155 mimic or inhibitor. All data were presented as mean ± SD; n = 3. ^##^ *P* < 0.01, ^###^ *P* < 0.001 *vs.* control; ^*^ *P* < 0.05, ^**^P < 0.01, ^***^P < 0.001 *vs.* LPS or IMQ-induced vehicle group.

To give insight into the changes of expression level of various miRNAs in psoriasis, we firstly predicted the conserved miRNAs with good mirSVR scores (≤−0.1) on *tnfaip3* mRNA 3′UTR and their proper target sites on miRanda (**Table S3**). Low mirSVR and high PhastCons make it more stable for combination of miRNA-mRNA and more conserved. Expression of 24 screened miRNAs in HaCaT cells were then detected by qRT-PCR following 24 h treatment with LPS. We found that miR-155 expression was substantially increased in LPS-induced cells (**Supplementary Figure S4**). To determine whether Ebosin could modulate miR-155 expression, we measured the miR-155 level *in vitro* and *in vivo*. As shown in **Figures 6A, B**, Ebosin was observed to decrease miR-155-3p expression in a dose-dependent manner both in LPS-induced HaCaT cells and in psoriatic mice. To investigate whether miR-155 directly binds to the 3′UTR of *tnfaip3*, it was cloned into a vector containing firefly luciferase reporter gene. Meanwhile, a mutant vector was constructed to eliminate the possible recognition by replacing nine seed nucleotides (TGTTAATGC to ACAATTACG) (**Figure 6C**). The result showed that the luciferase activity of *tnfaip3* 3′UTR was reduced (~40%) in the presence of miR-155 mimics (**Figure 6C**). However, all changes disappeared in cells transfected with the mutant vector. To further validate *tnfaip3* as a target of miR-155, the expression of A20 was shown to decrease significantly in HaCaT cells transfected with miR-155 mimics, while it was increased in blockage cells transfected with miR-155 inhibitor (**Figure 6D**). Taken together, these results indicate that *tnfaip3* is directly targeted by miR-155.

## Discussion

Ebosin is a novel exopolysaccharide isolated from metabolites of *Streptomyces* sp. 139. It exhibited *in vivo* inflammatory inhibition on both adjuvant arthritis and type II collagen arthritis rat model. Ebosin effectively reduced inflammation and joint damage through inhibiting proteins expression in MAPK and NF-κB signaling pathways *in vitro*, followed by production suppression of IL-1, TNFα, MMPs, and CC chemokines (25, 31). Excitingly, Ebosin could significantly suppress the production of CXCL-9 in preliminary experiments (**Supplementary Figures S1C, D**), which is a key indicator of psoriasis. From these results, we deduced that Ebosin has potential effects on psoriasis. Accordingly, RNA-Seq was conducted on LPS-induced HaCaT cell with or without Ebosin treatment, and it was found that Ebosin could decrease the expression of genes encoding for proteins within IL-17 signaling pathway and various cytokines as well as chemokines (**Supplementary Figure S2**).

Psoriasis is a multifactorial inflammatory skin disease characterized by keratinocyte hyperproliferation of epidermis, immune cell infiltration and disrupted epidermis barrier function. Driven by the exciting activity of Ebosin *in vitro*, we investigated the effect of Ebosin in IMQ-induced mice. Our study revealed that Ebosin alleviated IMQ-induced psoriasis-like skin inflammation *in vivo*. It improved skin thickening, erythema, and dramatically reduced the PASI score. mRNA levels of *krt1, krt10, s100a8*, and *s100a9* related to epidermal differentiation were decreased by Ebosin, indicating that Ebosin could reduce hyperplasia of epidermis by targeting the key cells.

**Figure 7:**
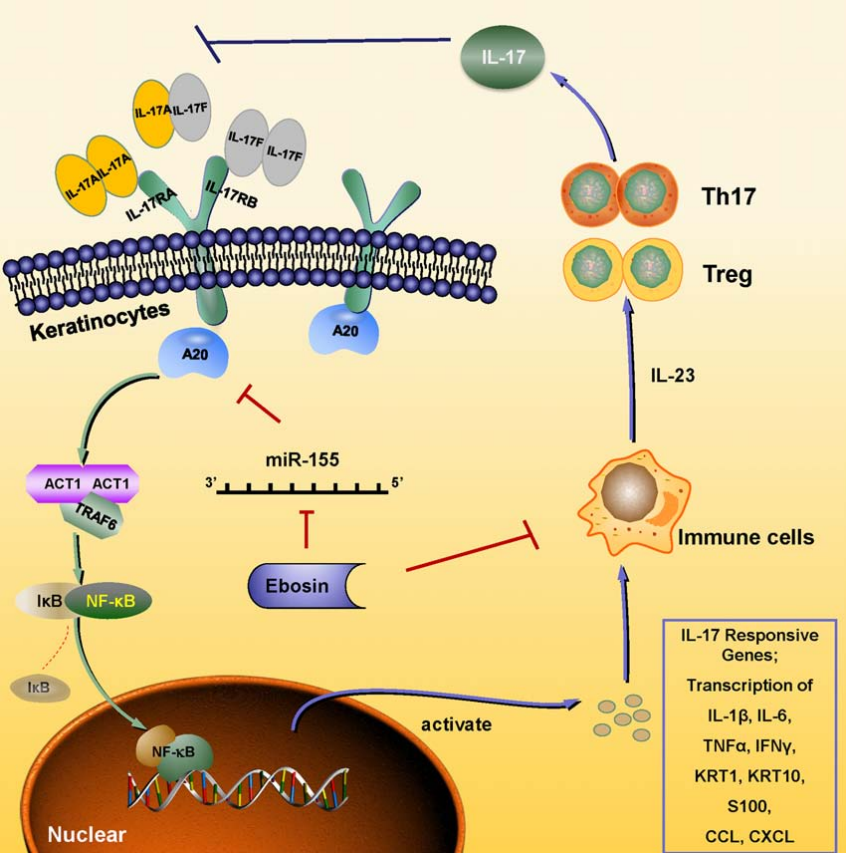
Schematic of the mechanism underlying the Ebosin inhibition of psoriasis development. Ebosin could decrease miR-155 expression, which is responsible for the regulation of A20, leading to the balance of Treg/Th17 and the decreased secretion of inflammatory cytokines *via* inactivation of NF-kB.

Except for hyperplasia of epidermal cells, immune cell infiltration plays a key role in the occurrence and development of psoriasis. The IMQ-induced psoriasis-like skin inflammation in mice has been found to be mediated *via* the IL-23/IL-17 axis (32). IL-23 activates Th17 cells through STAT3 pathway and promotes the production of cytokines, such as IL-17, IL-22, and TNF-α, and induces the proliferation of keratinocytes expressing IL-22 receptor (33, 34). Antagonists of IL-23 and IL-17 showed good therapeutic effects, which also validate the important role of the IL-23-IL-17 axis in psoriasis pathogenesis (35–38). In addition, CD4^+^CD25^+^Foxp3^+^ Tregs could differentiate into IL-17A–producing Th17 cells in psoriasis and aggravate its symptoms by inducing imbalance of Th17/Treg, activating effecting T cells and immune response in skin (39, 40). Our study revealed that Ebosin alleviated IMQ-induced psoriasis-like skin inflammation *in vivo* and linked to the balance of Treg/Th17 and IL-17 signaling pathway. Ebosin can reduce Act1, TRAF6, and phosphorylation of p65 in psoriatic mice. Act1 is an important adaptor of IL-17 family members, and its binding protein TRAF6 is ubiquitinated by Act1 to mediate MAPK and NF-κB pathway, leading to the upregulation of cytokines such as IL-23, IL-17, IL-22, IL-6, TNF-α, and CCL-20 (19, 41–43). More importantly, *tnfaip3* and *tnip1* genes play key roles to regulate NF-κB signaling in psoriasis, and Ebosin can increase the expression of A20, a key player in the negative feedback regulation of NF-kappaB signaling encoded by *tnfaip3*, which has been reported to attenuate NF-κB signaling pathway by editing the ubiquitination of proximal signaling proteins including TRAF6, MALT1, RIPK1, NEMO, UBCH5C, etc (44). It has been shown that lack of A20 impairs the NF-κB negative feedback loop and endorses PKC and p38 MAPK signaling, inducing expression of IL-17 (45). In this study, Ebosin increased the expression of A20, leading to the negative feedback of regulation of NF-kappaB and accumulation of Th17 cells, preventing IL-17A expression. Thus, it is indicated that Ebosin exerts antipsoriatic effects through regulating A20/NF-κB signaling pathway (**Figure 7**).

To further study how Ebosin regulates A20, we focus on the miRNA related psoriasis. MicroRNAs are short noncoding RNAs that could regulate the expression of protein-coding genes at the posttranscriptional level, and several miRNAs have been found effective on keratinocyte and immune cells function in psoriatic patients (46–48). MicroRNA 155 was found to be high expressed in autoimmune disease and was positively correlated with PASI and IL-17A level in psoriatic patients. It was reported that miR-155 level was influenced by transcription factor Foxp3, and its deletion could inhibit the immune response of CD4^+^ and CD8^+^ T cells, leading to the differentiation of Treg and Th17 cells by targeting SOCS1 (49, 50). In this study, miR-155 was found to be varied on LPS-induced HaCaT cells and recovered after Ebosin treatment both *in vitro* and *in vivo*. miR-155 was found to act on *tnfaip3* 3′UTR deduced on miRanda (http://www.miranda.org/) and was validated by luciferase assay. The induction and inhibition of miR-155 *in vitro* could directly down- and up-regulate the expression of A20. It is illustrated that miR-155 regulates Act1/TRAF6/NF-κB by targeting A20, and Ebosin regulates IL-17 pathway and Th17/Treg imbalance by miR-155 in psoriasis.

## Conclusions

Through RNA-Seq of HaCaT cells treated with Ebosin, we found that Ebosin can target the encoding genes of cytokines and IL-17 signaling pathway in epidermic cells. Further research demonstrated that Ebosin can ameliorate psoriatic skin lesions and reduce inflammatory factors expression *in vivo*. The protective role of Ebosin against inflammation-mediated psoriasis is through miR-155-*tnfaip3*-IL-17 axis. Compared with MTX, Ebosin significantly increased the percentage of CD4^+^Foxp3^+^CD25^+^ Tregs and decreased CD4^+^IL17A^+^ Th17 cells in psoriatic mice. Based on our results, we propose that Ebosin may act as a promising therapeutic agent against psoriasis *via* targeting epidermic and T cells.

## Data Availability Statement

The original contributions presented in the study are included in the article/**Supplementary Material**. Further inquiries can be directed to the corresponding authors.

## Ethics Statement

The animal study was reviewed and approved by Animal Experimental Ethics Committee of Institute of Medicinal Biotechnology, Chinese Academy of Medical Sciences & Peking Union Medical College.

## Author Contributions

WG and FX performed most of the research work. WG drafted the manuscript. ZZ and ZL devoted to ebosin preparation. JX and LB designed the research and revised the paper. All authors contributed to the article and approved the submitted version.

## Funding

This work was supported by grants from National Key Research and Development Program of China (2018YFA0902000), National Natural Science Foundation of China (81903632 and 31870059), and The Drug Innovation Major Project (2018ZX09711001-007-003).

## Conflict of Interest

The authors declare that the research was conducted in the absence of any commercial or financial relationships that could be construed as a potential conflict of interest.
