# Supplementary material for: Ebosin Ameliorates Psoriasis-Like Inflammation of Mice via miR-155 Targeting tnfaip3 on IL-17 Pathway
Source: Front Immunol. 2021 Apr 26;12:662362. doi: 10.3389/fimmu.2021.662362 (PMC8107364; doi:10.3389/fimmu.2021.662362)
Supplement: Supplementary file 1 [file DataSheet_1.docx]

**Supporting Information for**

**Original article**

**Ebosin ameliorates psoriasis-like inflammation of mice via miR-155 targeting *tnfaip3* on IL-17 pathway**

**
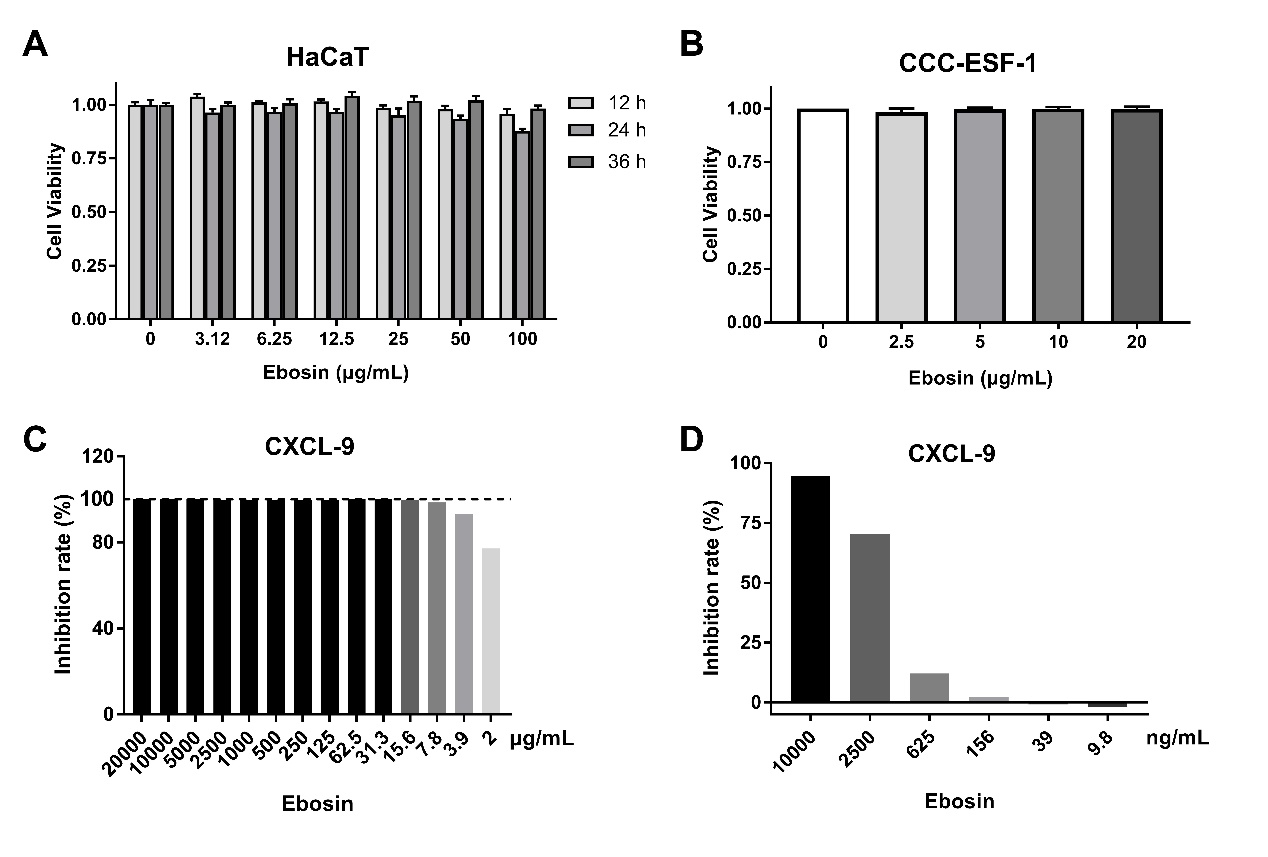
**

**Figure S1** The toxic effects of Ebosin on HaCaT cells (A) and CCC-ESF-1 cells (B). (C-D) CXCL-9 production of IFNγ-induced HaCaT cells by ELISA after treatment of Ebosin (mean ± SD, *n* = 3).


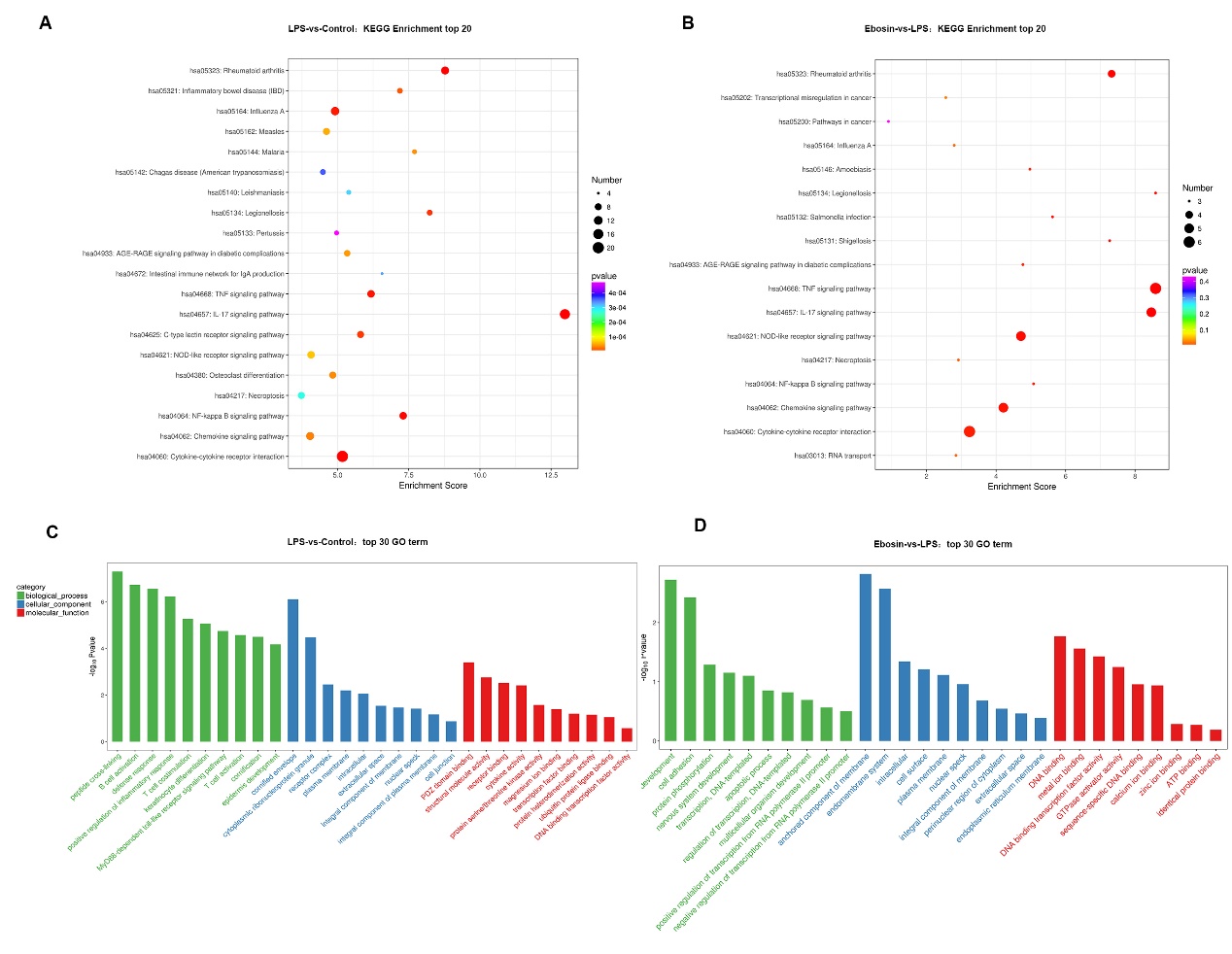


**Figure S2** KEGG and GO term of LPS-induced HaCaT cell. (A)KEGG analysis of RNA-seq for control vs. LPS-induced HaCaT cells. (B) KEGG analysis of RNA-seq for LPS-induced HaCaT cells without or with Ebosin. (C)GO analysis of RNA-seq for control vs. LPS-induced HaCaT cells. (D) GO analysis of RNA-seq for LPS-induced HaCaT cells without or with Ebosin.


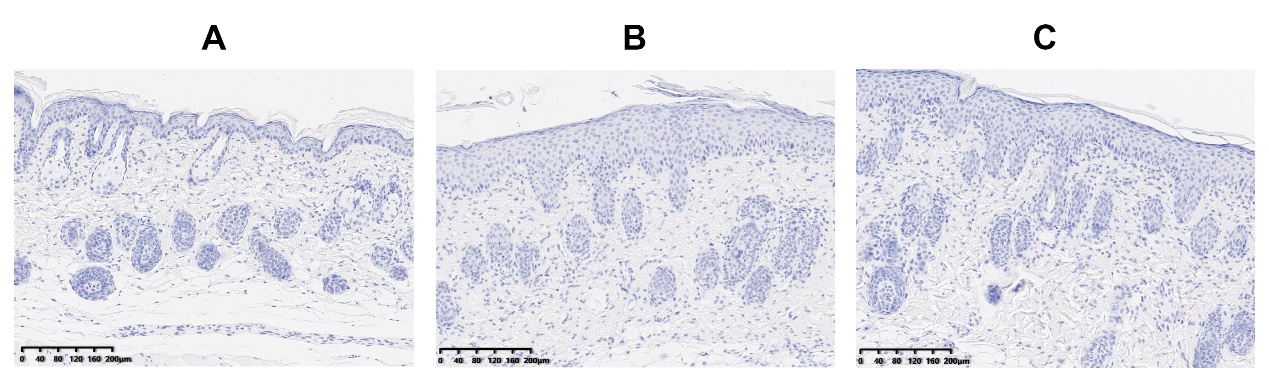


**Figure S3** Immunohistochemical images of the isotype control staining (magnification: 100X) for CD3 (A), CD8 (B) and Ki67 (C). The data suggested specific antibody staining is easily distinguished from background staining with the isotype control.


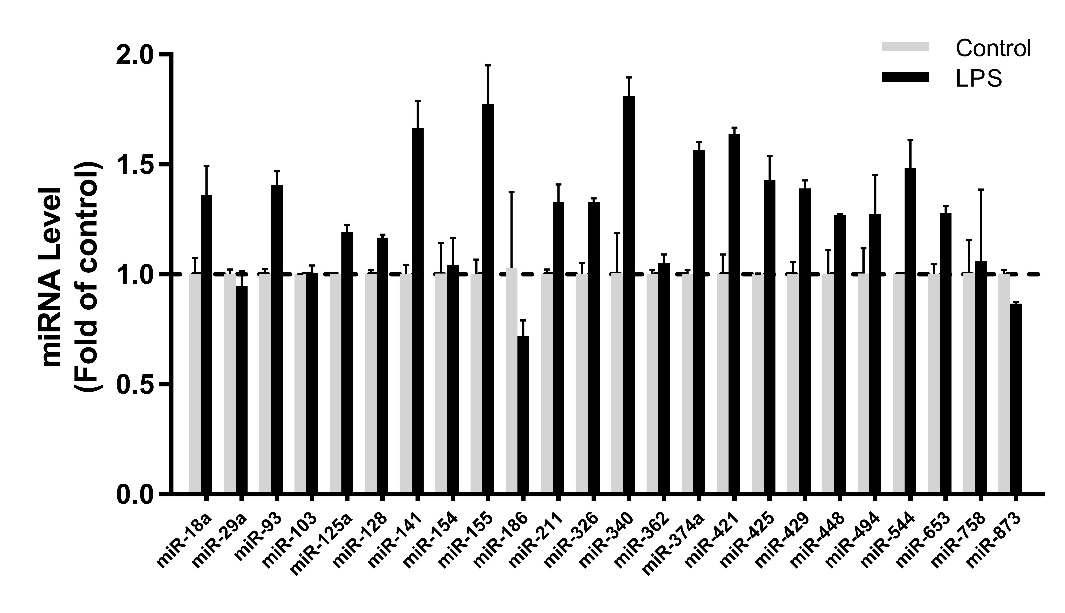


**Figure S4** RT-PCR analysis of the expression levels of various miRNAs in the LPS-induced HaCaT cells.

**Table S1.** The primers used for qRT-PCR

| Gene name | Forward primer (5’-3’) | Reverse primer (5’-3’) |
| --- | --- | --- |
| *h-ccl2* | CAGCCAGATGCAATCAATGCC | TGGAATCCTGAACCCACTTCT |
| *h-mmp13* | ACACCGTCAGCCGATTTGCTA | CCAAAGTAGACCTGCCCGGAC |
| *h-ccl20* | GGCTGCCTTCCTCTTC | TTTGCCAGTCACCTCTAA |
| *h-il-6* | ACTCACCTCTTCAGAACGAATTG | CCATCTTTGGAAGGTTCAGGTTG |
| *h-il-8* | ACTGAGAGTGATTGAGAGTGGAC | AACCCTCTGCACCCAGTTTTC |
| *h-gapdh* | GGAGCGAGATCCCTCCAAAAT | GGCTGTTGTCATACTTCTCATGG |
| *m-il-23* | AATAATGTGCCCCGTATCCAGT | GCTCCCCTTTGAAGATGTCAG |
| *m-il-17a* | TTTAACTCCCTTGGCGCAAAA | CTTTCCCTCCGCATTGACAC |
| *m-il-22* | ATGAGTTTTTCCCTTATGGGGAC | GCTGGAAGTTGGACACCTCAA |
| *m-tnip1* | CCAGGTACATCCTGCTACCAG | CGCCATTGGACGGAGATTTG |
| *m-tnfaip3* | ACCATGCACCGATACACGC | AGCCACGAGCTTCCTGACT |
| *m-tnfα* | ACACCGTCAGCGATTTGCTA | CCAAAGTAGACCTGCCCGGAC |
| *m-il-1β* | GCAACTGTTCCTGAACTCAACT | ATCTTTTGGGGTCCGTCAACT |
| *m-il-6* | AGTTGCCTTCTTGGGACTGA | CAGAATTGCCATTGCACAAC |
| *m-ccl20* | ACTGTTGCCTCTCGTACATACA | GAGGAGGTTCACAGCCCTTTT |
| *m-ccl2* | TAAAAACCTGGATCGGAACCAAA | GCATTAGCTTCAGATTTACGGGT |
| *m-s100a8* | AAATCACCATGCCCTCTACAAG | CCCACTTTTATCACCATCGCAA |
| *m-s100a9* | AGTGTCCTCAGTTTGTGCAG | ACTCCTTGTGGCTGTCTTTG |
| *m-krt10* | CGAAGAGCTGGCCTACCTAAA | GGGCAGCGTTCATTTCCAC |
| *m-krt1* | ATGACCAAAGTTGAGCTTCAGG | CAGATCCAAACTGCGGTTGTT |
| *m-gapdh* | AACAGCAACTCCCACTCTTC | CCTGTTGCTGTAGCCGTATT |

**Table S2.** The sequences of miRNA and the forward primers used for qRT-PCR

| miRNA | Sequence (5’-3’) | Forward primer (5’-3’) |
| --- | --- | --- |
| miR-18a-5p | taaggtgcatctagtgcagatag | taaggtgcatctagtgcaga |
| miR-29a-3p | tagcaccatctgaaatcggtta | tagcaccatctgaaatcgg |
| miR-93-5p | caaagtgctgttcgtgcaggtag | caaagtgctgttcgtgca |
| miR-103-3p | agcagcattgtacagggctatga | agcagcattgtacag |
| miR-125a-5p | tccctgagaccctttaacctgtga | tccctgagaccctttaacct |
| miR-128-3p | tcacagtgaaccggtctcttt | tcacagtgaaccggtct |
| miR-141-3p | taacactgtctggtaaagatgg | taacactgtctggtaaaga |
| miR-154-5p | taggttatccgtgttgccttcg | taggttatccgtgttgcctt |
| miR-155-3p | ctcctacatattagcattaaca | ctcctacatattagcattaaca |
| miR-186-5p | caaagaattctccttttgggct | caaagaattctccttttg |
| miR-211-5p | ttccctttgtcatccttcgcct | ttccctttgtcatccttcg |
| miR-326 | cctctgggcccttcctccag | cctctgggcccttcct |
| miR-340-5p | ttataaagcaatgagactgatt | tataaagcaatgagactg |
| miR-362-3p | aacacacctattcaaggattca | acacacctattcaaggattca |
| miR-374a | ttataatacaacctgataagtg | ttataatacaacctgataagtg |
| miR-421-3p | atcaacagacattaattgggcgc | atcaacagacattaattgggc |
| miR-425-5p | aatgacacgatcactcccgttga | aatgacacgatcactc |
| miR-429 | taatactgtctggtaaaaccgt | taatactgtctggtaaaaccgt |
| miR-448 | ttgcatatgtaggatgtcccat | ttgcatatgtaggatgtc |
| miR-493 | tgaaacatacacgggaaacctc | tgaaacatacacgggaa |
| miR-544 | attctgcatttttagcaagttc | attctgcatttttagcaagt |
| miR-653-5p | gtgttgaaacaatctctactg | gtgttgaaacaatctctactg |
| miR-758-3p | tttgtgacctggtccactaacc | tttgtgacctggtccacta |
| miR-873-5p | gcaggaacttgtgagtctcct | gcaggaacttgtgagtctc |

**Table S3.** miRanda-predicted target sites of conserved miRNAs with good mirSVR scores (≤-0.1) on *tnfaip3* mRNA 3’UTR

| hsa-miRNA | mirSVR score | PhastCons score |
| --- | --- | --- |
| hsa-miR-29a | -0.2981 | 0.4830 |
| hsa-miR-29b | -0.2981 | 0.4830 |
| hsa-miR-29c | -0.2981 | 0.4830 |
| hsa-miR-204 | -0.2451 | 0.4970 |
| hsa-miR-211 | -0.2473 | 0.4970 |
| hsa-miR-125a-5p | -0.2577 | 0.4970 |
| hsa-miR-125b | -0.2471 | 0.4970 |
| hsa-miR-544 | -0.1298 | 0.5220 |
| hsa-miR-340 | -1.0451 | 0.5980 |
| hsa-miR-448 | -0.8561 | 0.5980 |
| hsa-miR-18a | -1.1640 | 0.5980 |
| hsa-miR-18b | -1.1640 | 0.5980 |
| hsa-miR-329 | -0.1051 | 0.5966 |
| hsa-miR-362-3p | -0.1083 | 0.5966 |
| hsa-miR-103 | -0.7482 | 0.6164 |
| hsa-miR-107 | -0.7482 | 0.6164 |
| hsa-miR-154 | -0.7643 | 0.5452 |
| hsa-miR-211 | -0.2519 | 0.5566 |
| hsa-miR-204 | -0.2519 | 0.5566 |
| hsa-miR-93 | -0.2203 | 0.5680 |
| hsa-miR-519d | -0.2183 | 0.5680 |
| hsa-miR-106a | -0.2183 | 0.5680 |
| hsa-miR-106b | -0.2183 | 0.5680 |
| hsa-miR-17 | -0.2183 | 0.5680 |
| hsa-miR-20a | -0.2183 | 0.5680 |
| hsa-miR-20b | -0.2183 | 0.5680 |
| hsa-miR-200a | -0.1798 | 0.5680 |
| hsa-miR-141 | -0.1798 | 0.5680 |
| hsa-miR-873 | -0.1905 | 0.4947 |
| hsa-miR-494 | -0.1031 | 0.5180 |
| hsa-miR-98 | -0.1067 | 0.5441 |
| hsa-miR-128 | -0.4162 | 0.5677 |
| hsa-miR-425 | -0.7561 | 0.5566 |
| hsa-miR-326 | -0.4472 | 0.5171 |
| hsa-miR-330-5p | -0.4472 | 0.5171 |
| hsa-miR-340 | -0.9682 | 0.5171 |
| hsa-miR-200a | -0.9287 | 0.5171 |
| hsa-miR-141 | -0.9287 | 0.5171 |
| hsa-miR-421 | -0.1840 | 0.7145 |
| hsa-miR-23a | -1.2941 | 0.7145 |
| hsa-miR-23b | -1.2941 | 0.7145 |
| hsa-miR-340 | -0.3736 | 0.7076 |
| hsa-miR-374a | -1.2804 | 0.7007 |
| hsa-miR-374b | -1.2887 | 0.7007 |
| hsa-miR-429 | -0.1837 | 0.7007 |
| hsa-miR-200b | -0.1820 | 0.7007 |
| hsa-miR-200c | -0.1820 | 0.7007 |
| hsa-miR-19a | -1.3099 | 0.7007 |
| hsa-miR-19b | -1.3099 | 0.7007 |
| hsa-miR-455-5p | -0.2414 | 0.7007 |
| hsa-miR-186 | -0.6826 | 0.6969 |
| hsa-miR-758 | -0.1218 | 0.6930 |
| hsa-miR-653 | -0.2001 | 0.6930 |
